# The Longevity of Th2 Humoral Response Induced by Proteases Natterins Requires the Participation of Long-Lasting Innate-Like B Cells and Plasma Cells in Spleen

**DOI:** 10.1371/journal.pone.0067135

**Published:** 2013-06-28

**Authors:** Evilin Naname Komegae, Lidiane Zito Grund, Monica Lopes-Ferreira, Carla Lima

**Affiliations:** 1 Immunoregulation Unit, Special Laboratory of Applied Toxinology, Butantan Institute, São Paulo, Brazil; 2 Department of Immunology, University of São Paulo, São Paulo, Brazil; King's College London, United Kingdom

## Abstract

The generation of long-lived antibody-secreting cells (ASC) and memory B cells are critical events for an effective vaccine and the choice of adjuvant can influence these processes. Various cellular and molecular mechanism involved in the protease action that determine Th2 responses have been identified. However, direct or indirect actions in the regulation of the induction, survival and longevity of ASC in differential compartments remain largely unknown. We investigated whether the proteolytic activity of proteins are determinant for the modulation of the memory immune response in mice, promoting the differentiation of memory B cells to terminally differentiated end stage cells. Here, we show that the proteolytic activity of Natterins, from the venom of *Thalassophryne nattereri* Brazilian fish, besides inducing a Th2 response with plasmatic titers of high-affinity antigen-specific IgE over extended periods is sufficient for the generation of signals that contribute to the formation of a survival niche in the spleen, essential for the longevity of the main subtype of ASC with B220^neg^ phenotype.

## Introduction

Type I IgE-mediated hypersensitivity reactions, a classical prototype Th2 polarized response, are initiated by the recognition of allergens by dendritic cells (DC), and culminate in Th2 cell differentiation, IgE antibodies (Abs) production, and mast cell sensitization and triggering. Part of the peripheral B cell compartment has undergone class switching to IgE. The class switch to IgE is a tightly regulated process that requires a Th2-biased cytokine milieu and a concerted series of gene rearrangements and splicing events.

Because long-lived antibody-secreting cells (ASC) are the source of IgE, recruitment and selection of memory B cells (Bmem) into the ASC compartment is a critical step in immune deregulation that leads to the production of IgE. Recent data [1]–[3] suggest a role of murine IgE-producing splenic memory B cell and ASC in the development and maintenance of allergies.

Two B cell populations are responsible for sustaining the humoral immune memory: Bmem and ASC [4]. Memory B cells undergo rapid clonal expansion and differentiation to mount high affinity Abs response upon exposure to antigens. ASC (positive for syndecan-1– CD138) are terminally differentiated and continue secreting high affinity antigen-specific Abs for protracted periods of time without antigenic stimulation in the bone marrow (BM) that provides a special microenvironment for their longevity [5].

Both Bmem and ASC can be generated during the first immune response from innate-like B cells as B1 and follicular and marginal conventional B (B2). Apart from their functional specialization, anatomical location, and self-renewing capacity, B1 cells can be distinguished from the more abundant B2 cells by their surface markers, because they are CD23^neg^, B220^low^, and IgM^high^ [6].

A number of environmental allergens from diverse sources have proteinase activity, which has been suggested to skew the immune response toward the Th2 phenotype. Although all described allergenic proteinases differ, lacking structural motifs that uniquely induce Th2 and IgE responses, the role of their proteinase activity in the development of allergic sensitization has been shown for cockroach frass [7]–[8], house dust mite [9]–[10], recombinant *Leishmania mexicana* CP virulence factor [11], and fungal allergens [12]–[13]. The literature on the protease derived from allergens is extensive, and its role for development of Th2 polarized responses is well established [14].

Whether proteases of venomous fish are optimal for strong and IgE Abs response has, however, not been investigated. Moreover, the direct or indirect actions of fish proateases in the regulation of the induction, survival and longevity of Ab-producing Bmem or ASC in differential compartments have not been addressed.

Recently, a group of toxins in the venom of *Thalassophryne nattereri* Brazilian fish, denominated Natterins, was characterized and identified as proteases with kininogenase activity [15]–[16]. Natterins (1, 2, 3, 4 and P) cleave the human kininogen and have molecular mass around 38 kDa and are the major toxins responsible for nociception and edema induced in mice by the venom [17]. We also reported that immunization with lower doses of venom induced a strong long-lasting B-cell memory against venom antigens with persistent levels of IL-5 and of IgG1 and IgE Abs [18]. Furthermore, we have provided evidence that IL-17A as well as IL-5 produced in a context of chronic inflammatory response against venom proteins directly influence the production of specific-IgE Abs and the retention of B1a cells in the BM from the spleen. Both cytokines negatively regulate the maintenance of ASC B220^pos^ in different sites of response. And a striking finding in this study was that IL-5 and IL-17A are critical for the differentiation and survival of ASC with B220^neg^ phenotype in inflamed peritoneal cavity [19]. Therefore, this model provides an interesting scenario for studying the signals allowing survival and differentiation of the memory B cells compartment.

These observations raise the question of whether the protease activity of Natterins would bias the immune response in favor of IgE and modulate the compartment of innate-like B cells and memory B cells, inducing the differentiation of long-lived antibody-secreting cells terminally-differentiated. Therefore, we examined the secondary immune response elicited by Natterins, focusing on the expansion of innate-like B cells and memory B cells upon antigen recall *in vivo*, the subtypes of ASC with B220^neg/pos^ phenotypes in different compartments, and the specific-Abs production. Our study indicates that the proteolytic activity of Natterins besides inducing a Th2 response with plasmatic titers of high affinity antigen-specific IgE over extended periods is sufficient for the generation of survival signals that contribute to the formation of a molecular survival niche in the spleen, essential for the longevity of the ASC with B220^neg^ phenotype.

## Materials and Methods

### Mice

Male BALB/c mice (5–6 weeks old) were obtained from a colony at the Butantan Institute, São Paulo, Brazil. Animals were housed in a laminar flow holding unit (Gelman Sciences, Sydney, Australia) in autoclaved cages on autoclaved bedding, in an air-conditioned room in a 12 h light/dark cycle. Irradiated food and acidified water were provided *ad libitum*. This study was carried out in strict accordance with the recommendations in the Guide for the Care and Use of Laboratory Animals of the Brazilian College of Animal Experimentation. The protocol was approved by the Committee on the Ethics of Animal Experiments of the Butantan Institute (Permit Number: 504/08) and of University of São Paulo (Permit Number: 747/10). All surgery was performed under sodium pentobarbital anesthesia, and all efforts were made to minimize suffering.

### Natterins Preparation

*T. nattereri* fish venom was obtained from fresh captured specimens at the Mundau Lake in Alagoas, state of Brazil with a trawl net from the muddy bottom of lake. No protected specimens were captured and fish were transported to Immunoregulation Unit of Butantan Institute. All necessary permits were obtained for the described field Studies (capture, conservation and venom collection - IBAMA Permit Number: 16221-1). Venom was immediately extracted from the openings at the tip of the spines by applying pressure at their bases. After centrifugation, venom was pooled and stored at −80°C before use. After that fish were anesthetized with 2-phenoxyethanol prior to sacrifice by decapitation. The purified 38-KDa Natterins from *T. nattereri* fish venom was prepared with a pool of venom collected in different months of the year in the state of Alagoas according to [17]. The venom was fractionated by cation exchange chromatography, using the fast protein liquid chromatography system (FPLC - Pharmacia, Uppsala, Sweden). Immediately before chromatography, 2 mg venom was diluted in 500 µL of buffer A (20 mM Tris-hydroxymethylaminomethane, pH 8.3) and the solution centrifuged at 10.000 g for 5 min. The sample was applied on Mono S column HR 5/5 equilibrated with buffer A. The retained proteins are eluted with a linear gradient of NaCl (sodium chloride) 0–2 M and collected at a flow rate of 1.0 mL/min. The elution profile is determined by measuring absorbance at 280 nm. Fractions 1–4, except the fifth, corresponding to Natterins were pooled, dialyzed against 50 mM Tris/HCl pH 7.4 and evaluated with respect to its protein content and kept at −20°C until use. Natterins obtained were analyzed by polyacrylamide gel electrophoresis with 12% SDS (SDS-PAGE) according to Laemmli [20]. Endotoxin content was evaluated (resulting in a total dose <0.8 pg LPS) with QCL-1000 chromogenic Limulus amoebocyte lysate assay (Bio-Whittaker) according to the manufacturer’s instructions.

The total amount of protease was determined using quenched fluorescein-kininogen substrate (Molecular Probes). Enzymatic release of fluorescent signals was quantified by a microplate fluorometer BMG Fluostar Galaxy V4.30.0 (BMG Labtechnologies) according to the manufacturer’s instructions and data was expressed as percentage of inhibition [17]. In some experiments, the proteolytic activity of Natterins was blocked by metal-chelating agents (EDTA and o-PHE) according to Lopes-Ferreira et al. [17], or by heat inactivation at 65°C for 30 min according to Arizmendi and colleagues [8]. The loss of enzymatic activity by both treatments was monitored by quenched fluorescein-kininogen substrate. In contrast, PMSF, TLCK, TPCK, E-64, and Pepstatin-A did not present inhibitory effect on enzymatic activity of Natterins.

### Induction of Memory Immune Response by Natterins

Groups of 5 mice were immunized with i.p. injections of 10 µg of active or inactivated Natterins on day 0 and boosted on days 7, 21 and 28. The first immunization was given in 1.6 mg of aluminum hydroxide (Al(OH)_3_) as adjuvant. Animals injected on day 0 only with adjuvant were considered as the control group. Blood samples were collected at 48, 74, and 120 d after the first immunization and stored at –20°C until analysis. After that mice were killed by the injection of lethal dose of sodium pentobarbital anesthesia, and peritoneal fluid, spleen and BM were harvested at various time points, and single cell suspensions were prepared as described previously [21].

Separate groups of active Natterins-immunized Balb/c mice were tested on day 28 by for analysis of isotype switching according to Wesemann et al. [22]. Cells from peritoneal cavity, spleen, and BM were pelleted after surface staining (anti–B220-PE-Cy5, anti-CD138 APC) and resuspended with 1 ml of 3% formaldehyde for 20 min at 37°C, followed by a wash with 2 ml of PBS and permeabilization with 90% cold methanol for 30 min on ice, washed twice with PBS. The cells were pelleted and resuspended in 1 ml of 0.5% saponin or 0.05% trypsin for 2 min in PBS at room temperature and incubated for 1 h with a cocktail of PE anti-IgG, and FITC anti-IgE antibodies (both from BioLegend). Cells were then washed and re-suspended for flow cytometry (Fig. S1.).

### Cell Preparation

The cells were obtained at 48, 74 and 120 d after the first immunization of mice and. Peritoneal cells were obtained by wash with 5 mL of the RPMI 1640 medium. Spleens were dissociated into single cell suspensions by mechanical disruption in cell strainer (BD Falcon). Bone marrow cells were obtained by flushing femurs of the mice. Erythrocytes in spleens and BM were lysed with 0.14 M NH_4_Cl and 17 mM Tris-Cl (pH 7.4). The identification of differential subtypes of innate-like B cells or memory B cells and ASC was conducted as described earlier [19].

### Flow Cytometry Analysis

For surface staining single-cell suspensions were treated with 3% mouse serum of naive mice to saturate Fc receptors followed by the staining by fluorescence conjugated Abs: Rat IgG2bk FITC-anti-mouse CD3, Armenian hamster IgG1y2 FITC-anti-mouse CD19, Rat IgG2ak PE-anti-mouse CD5, Rat IgG2ak PE-anti-mouse CD23, Goat IgG2bk PE-anti-mouse IgG [specific for IgG1, IgG2a, IgG2b and IgG3], Rat IgG2bk PE-anti-mouse CD11b, Rat IgG2ak PerCP-Cy5-anti-mouse CD45R/B220, Rat IgG2ak FITC-anti-mouse CD43, and Rat IgG2ak PE-anti-mouse CD138 for 30 min on ice. Cells were washed three times in PBS 1% BSA. Negative controls were used to set the flow cytometer photomultiplier tube voltages, and single-color positive controls were used to adjust instrument compensation settings. The cells were examined for viability by flow cytometry using forward/side scatter characteristics or 7-AAD exclusion. Data from stained samples were acquired using a four-color FACSCalibur flow cytometer equipped with CellQuest software (BD Biosciences) and were analyzed using CellQuest Software (Becton-Dickinson, San Jose, CA).

### Titration of Total IgE and Specific-IgG1 and IgG2a by ELISA

Blood samples were obtained by the puncture of the right ventricle of immunized-mice. ELISA for detection of Abs was performed as described [23]. Microtitre 96-well plates (3590 - Costar, Cambridge, MA, USA) were coated with Natterins at 3 µg/mL or BSA (1 µg/mL) (negative control) in PBS for 18 h at 4 ^o^C. For blocking, the wells were washed with PBS, and incubated with 200 µL of 10% BSA in PBS for 3 h at 37 ^o^C. After washing, plasma were tested for IgG1 or IgG2a Abs using biotinylated goat anti-mouse IgG1 (31236) or IgG2a (31237) antiserum from Thermo Scientific Pierce (Rockford, IL USA) for 1 h at room temperature. The reactions were developed with streptavidin-horseradish peroxidase complex (Sigma), O-phenylenediamine (OPD) and H_2_O_2_ and the plates were read at 490 nm on an automated ELISA reader (Spectramax, Molecular Devices). The results were expressed as the mean ± SEM absorbance. An IgE-specific ELISA was used to quantitate total IgE Abs level in plasma using matched Abs pair (553413 Pharmingen and 1130-08 Southern Biotech.), according to the manufacturer’s instructions. Samples were quantified by comparison with a standard curve of IgE (0313ID, Pharmingen).

### Evaluation of Anaphylactic IgG1 and IgE by Passive Cutaneous Anaphylaxis (PCA)

The anaphylactic activity of IgG1 was evaluated by PCA reactions in mice as described by Ovary [24]. Mice were previously shaved and injected intradermically (50 µL) with three serial dilutions of plasma (inactivated for 1 h at 56°C) in each side of the dorsal skin. For IgE titration, PCA reactions were performed in rats using non-inactivated plasma, according to Mota and Wong [25]. After 2 or 18 h they were challenged i.v. with 50 or 100 µg of Natterins +0.25% of Evans blue solution. Extravasation of Evans blue dye, due to increased blood vessel permeability during the first 30 min of the PCA reactions is dependent mainly on histamine and serotonin released from activated mast cells. All tests were made in triplicate and PCA titers were expressed as the reciprocal of the highest dilution that gave a lesion of >5 mm in diameter. The detection threshold of the technique was established at 1∶5 dilutions.

### Statistical Analysis

All values were expressed as mean ± SEM. Experiments were performed three times. Parametric data were evaluated using analysis of variance, followed by the Bonferroni test for multiple comparisons. Non-parametric data were assessed using the Mann-Whitney test. Differences were considered statistically significant at *p*<0.05. The SPSS statistical package (Release 13.0, Evaluation Version, 2004) was employed.

## Results

### The Proteolytic Activity of Natterins is Essential for the Generation of Specific-Th2 Humoral Memory Response

The presence of protease activities in different allergens has been linked to allergic inflammation and a Th2 phenomenon. Atopic individuals have long been known to have the predilection to making antigen-specific Abs (IgE and IgG) against environmental allergens that can mediate hypersensitivity reactions. Studies indicate that proteolytically active molecules have the ability to induce IgE synthesis and consequent allergic response [26]–[29], [10]. Recently, a group of toxins in the venom of *T. nattereri*, denominated Natterins, was characterized and identified as proteases with kininogenase activity [16]. Natterins (1, 2, 3, 4 and P) cleave in an *in vitro* assay the human kininogen and have molecular mass around 38 kDa and are the major toxins responsible for nociception and edema induced in mice by the venom [17]. To explore the allergenic potential of Natterins with proteolytic activity, we performed experiments with immunized-mice with active or inactivated Natterins and compared the serum Ab titers.

**Figure 1:**
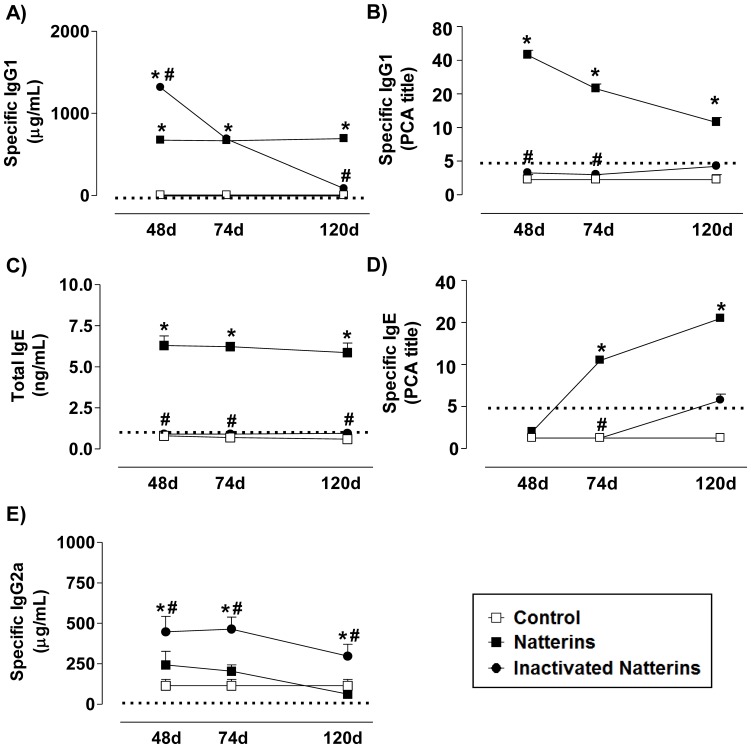
The proteolytic activity of Natterins is sufficient for the generation of long-lasting Th2 polarized humoral response. BALB/c mice were immunized with active or inactivated Natterins on day 0 and boosted 7, 21 and 28 days later. Serum anti-Natterins titers were determined by ELISA (A, C, and E). The anaphylactic IgG1 and IgE Abs were examined by PCA (B and D). Each group consisted of at least five male mice, and representative data from three repeated experiments are shown. *p<0.05 compared to control-mice, and #*p*<0.05 compared to active Natterins. The dotted line represents the Abs level of control non-immunized mice before immunization with adjuvant at day 0.

Intraperitoneal immunization of mice with proteolytically active Natterins induced a polarized Th2 responses with great amount of specific-IgG1 (Fig. 1A) until 120 d after immunization, and elevated titers of anaphylactic specific-IgG1 (Fig. 1B). Active Natterins caused unspecific polyclonal activation of B cells, inducing the production of negligible levels of total-IgE in serum (Fig. 1C). In addition, we observed the production of moderate titers of anaphylactic specific-IgE (Fig. 1D) until 120 d, but low negligible amounts of specific-IgG2a (Fig. 1E). We also found that inactivation of protease activity abolished the ability of the Natterins to induce persistent production of specific-IgG1 (Fig. 1A), total-IgE (Fig. 1C) and mainly the production of anaphylactic Abs as IgG1 (Fig. 1B) and IgE (Fig. 1D), favoring the development of specific Abs of the IgG2a subtype, characteristic of Th1 response. Control mice only immunized with adjuvant at day 0 did not produce any type of specific-Abs. Our results are direct evidence that the proteolytic activity of Natterins, proteases from the venom of *T. nattereri* is sufficient for the generation of long-lasting Th2 polarized humoral response.

### The Proteolytic Activity of Natterins Sustains the Chronic Persistence of Innate-like B Cells in the Spleen

Both Bmem and ASC can be generated during the first immune response from innate-like B cells as B1 and follicular and marginal conventional B (B2). We further examined the requirement of the proteolytic activity of Natterins to mediate B1 and B2 cell responses. We analyzed these populations by flow cytometer in cell suspensions of peritoneum cavity, spleen, and BM from active or inactivated Natterins-immunized mice. B1 and B2 cells can be distinguished in mice by the expression of traditional B cell markers (B1 cells are B220^pos^CD23^neg^, whereas B2 cells are B220^pos^CD23^high^). The mature B1a subset that predominates in the peritoneal and pleural cavities of mice has been defined as B220^pos^CD5^pos^IgM^high^IgD^how^, and an otherwise similar CD5^neg^ subset has been designated B1b [30].

**Figure 2:**
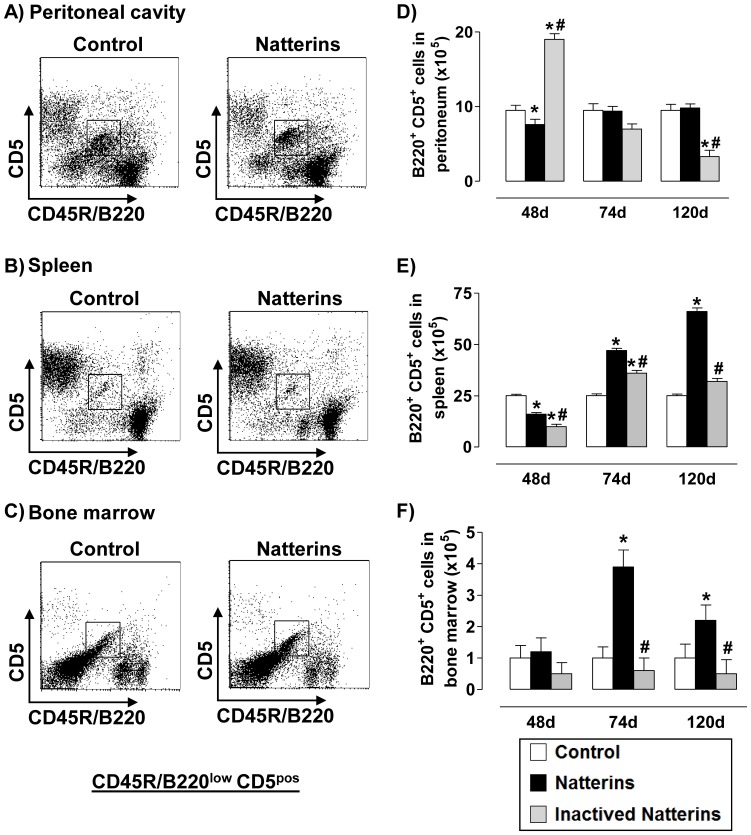
The proteolytic activity of Natterins drives the chronic maintenance of B1a cells in spleen and in bone marrow. A representative dot plot of B1a cells (B220^pos^CD5^pos^ from CD3-negative gated cells) obtained at 48, 74 and 120 d after immunization with active or inactivated Natterins is shown in peritoneum (***A***, ***D***), spleen (***B***, ***E***) and BM (***C***, ***F***). The bars representative of the absolute numbers of B220^pos^CD5^pos^ cells were determined from total mononuclear cells by multiparametric flow cytometer using Rat IgG2bk FITC-anti-mouse CD3, Rat IgG2ak PE-anti-mouse CD5, and Rat IgG2ak PerCP-Cy5-anti-mouse CD45R/B220. *p<0.05 compared to control-mice, and # *p*<0.05 compared to active Natterins.

A representative dot plot of B1a cells (B220^low^CD5^pos^ from CD3-negative gated cells) is showed for each compartment: the inflamed peritoneal cavity, favorable niche for repeated antigen encounter by the B cell receptor (Fig. **2** ***A***); in the spleen, an important secondary lymphoid organ (Fig. **2** ***B***), and in BM that contains survival niches for B cells (Fig. **2** ***C***). Our results showed that mice immunized with proteolytic active Natterins presented an increased number of B1a cells at 74 and 120 d in spleen (Fig. **2** ***E***) and in BM (Fig. **2** ***F***), but not in peritoneal cavity (Fig. **2** ***D***), compared with control mice that maintain basal level of cells in all compartments. In contrast, inactivated Natterins induced an increased number of B1a cells in the peritoneal cavity only at 48 d (Fig. **2** ***D***), and few cells in spleen at 74 d (Fig. **2** ***E***). These results suggest that the proteolytic activity of Natterins negatively controls the early expansion of B1a in the peritoneal cavity of mice. And in response to Natterins the persistence of these cells in the spleen and BM is totally dependent on its proteolytic activity.

**Figure 3:**
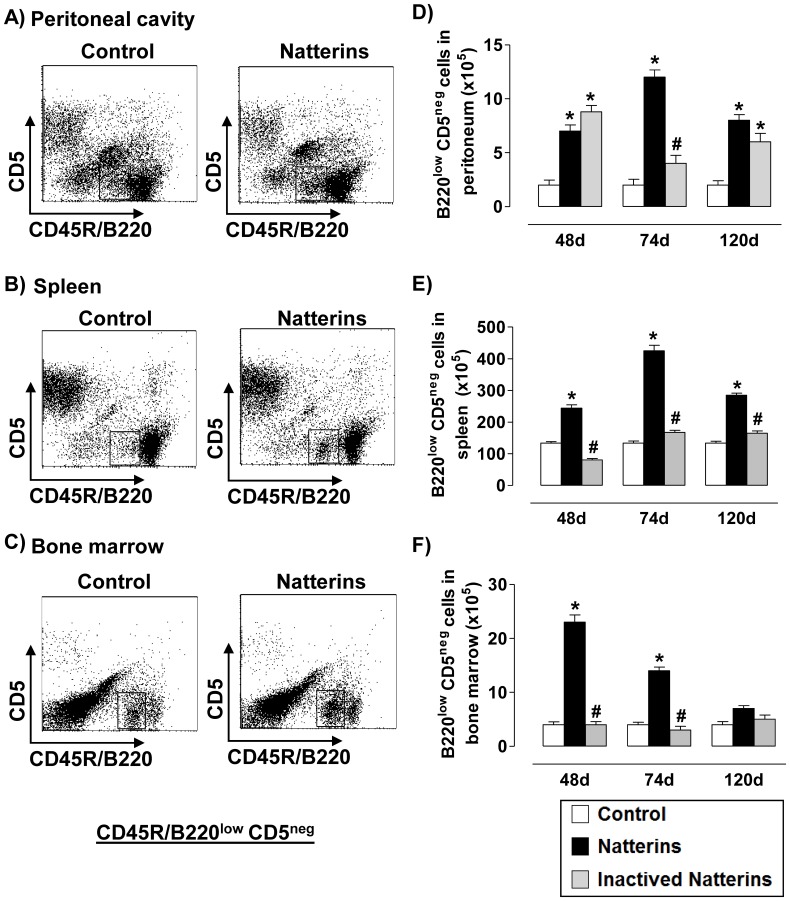
Natterins induce the B1b cell response. A representative dot plot of B1b cells (B220^pos^CD5^neg^ from CD3-negative gated cells) obtained at 48, 74 and 120 d after immunization with active or inactivated Natterins is shown in peritoneum (***A***, ***D***), spleen (***B***, ***E***) and BM (***C***, ***F***). The bars representative of the absolute numbers of B220^pos^CD5^neg^ cells were determined from total mononuclear cells by multiparametric flow cytometer using using Rat IgG2bk FITC-anti-mouse CD3, Rat IgG2ak PE-anti-mouse CD5, and Rat IgG2ak PerCP-Cy5-anti-mouse CD45R/B220. *p<0.05 compared to control-mice, and # *p*<0.05 compared to active Natterins.

Our results of B1b cells (B220^low^CD5^neg^ from CD3-negative gated cells) showed that the proteolytic activity of Natterins sustained elevated number of B1b cell in peritoneal cavity (Fig. **3** ***A and 3D***) and in spleen (Fig. **3** ***B and 3E***) until 120 d, and until 74 d in the BM (Fig. **3** ***C and 3F***) compared with control mice that maintain basal level of cells in all compartments. Inactivated Natterins sustained an elevation in the number of B1b cells in peritoneal cavity (Fig. **3** ***D***), while in spleen and in BM these cells were in the same number compared with control mice. Together, the proteolytic activity of Natterins positively controls the chronic retention of the largest absolute number of B1b cell in spleen.

**Figure 4:**
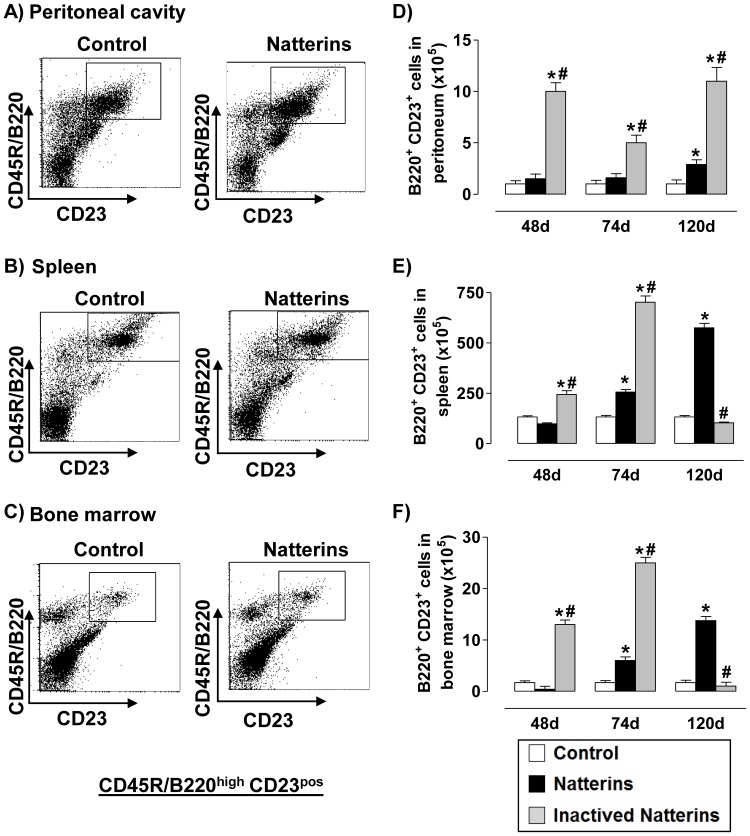
The chronic maintenance of B2 cells in spleen and in the bone marrow is dependent on proteolytic activity of Natterins. A representative dot plot of B2 cells (B220^pos^CD23^pos^ from CD3-negative gated cells) obtained at 48, 74 and 120 d after immunization with active or inactivated Natterins is shown in peritoneum (***A***, ***D***), spleen (***B***, ***E***) and BM (***C***, ***F***). The bars representative of the absolute numbers of B220^pos^CD23^pos^ cells were determined from total mononuclear cells by multiparametric flow cytometer using Rat IgG2bk FITC-anti-mouse CD3, Rat IgG2ak PE-anti-mouse CD23, Rat IgG2ak PerCP-Cy5-anti-mouse CD45R/B220. *p<0.05 compared to control-mice, and # *p*<0.05 compared to active Natterins.

Inactivated Natterins induced an increased response of B2 cells (B220^high^CD23^high^ from CD3-negative gated cells) in the spleen (Fig. **4** ***B and 4E***) and in the BM (Fig. **4** ***C and 4F***) until 74 d that was maintaining until 120 d in the inflamed peritoneal cavity (Fig. **4** ***A and 4D***). In contrast, the proteolytic activity of Natterins induced an increased number of B2 cells in all compartments mainly in the chronic phase of response (120 d). Basal levels of B2 cells were observed in control mice. These results suggest that the proteolytic activity of Natterins could contribute to the exit of B2 cells from the peritoneal cavity and for the chronic retention of B2 cells in spleen (largest absolute number) and in BM.

### The Proteolytic Activity of Natterins Regulates the Chronic Maintenance of Memory B Cell and ASC B220^neg^ in the Spleen

Upon activation by antigen in peripheral lymphoid organs, mature B2 cells may undergo IgH CSR, a process in which the IgH µ constant region exons (Cµ) are deleted and replaced by one of several sets of downstream CH exons (e.g., Cγ, Cε, and Cα), termed CH genes. Because Natterins activates B2 cells, and this cell type can contribute to GC reactions or become ASC in the presence of TLR agonists or infections [31], [6], we next examined whether Natterins induces GC memory B cell and plasma cell (ASC with positive or negative expression of B220) development.

**Figure 5:**
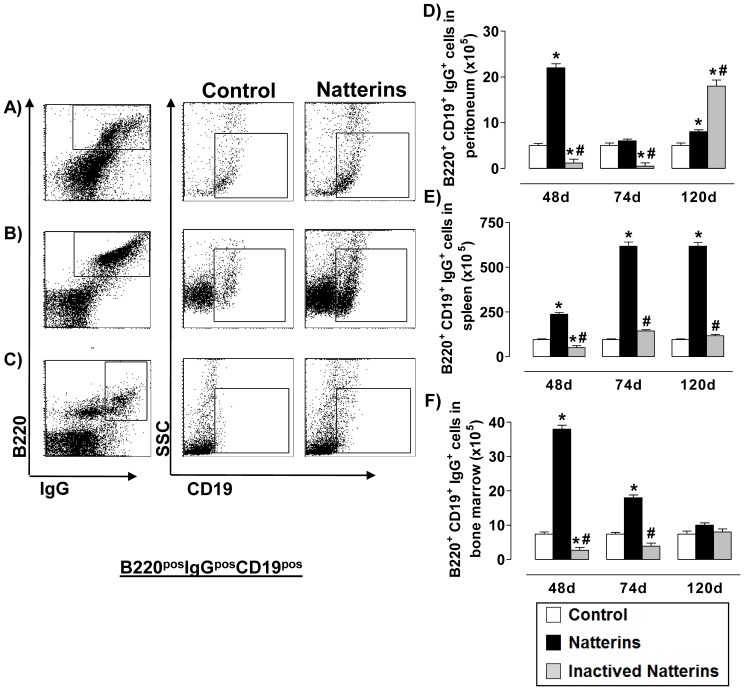
The proteolytic activity of Natterins is crucial for the maintenance of memory B cells in the spleen. Total memory B cell (CD19^pos^ in B220^pos^IgG^pos^ gated cells) numbers in peritoneum (***A***, ***D***), spleen (***B***, ***E***) and BM (***C***, ***F***) was determined from total mononuclear cells by multiparametric flow cytometer using Armenian hamster IgG1y2 FITC-anti-mouse CD19, Goat IgG2bk PE-anti-mouse IgG (specific for IgG1, IgG2a, IgG2b and IgG3), Rat IgG2ak PerCP-Cy5-anti-mouse CD45R/B220. *p<0.05 compared to control-mice, and # *p*<0.05 compared to active Natterins.

In the Figure **5**, we observed in active Natterins-immunized mice an elevated number of Bmem [CD19^pos^ in a gate of B220^pos^ x IgG^pos^ cells _ cell surface expression of switched IgG (IgG1, IgG2a, IgG2b and IgG3)] in the peritoneal cavity at 48 d (Fig. **5** ***A and 5D***) and in the BM until 74 d (Fig. **5** ***C and 5F***), but in the spleen elevated number of these cells was observed until 120 d (Fig. **5** ***B and 5E***). Switched Bmem were sustained in low levels in control mice during all time. The inactivation of protease abolished the ability of Natterins to induce this subpopulation in peritoneal cavity and in BM at 48 and 74 d, and also in the spleen during all time points. In contrast, inactivated Natterins intensified the expansion of B220^pos^CD19^pos^IgG^pos^ Bmem in peritoneal cavity at 120 d after immunization. These results suggest that the proteolytic activity of Natterins is determinant for the exit of Bmem from the peritoneal cavity and for the chronic maintenance in the spleen.

The cellular differentiation of Bmem into ASC has not been completely elucidated, but a hierarchical model of differentiation has been proposed: activated B cells progressively acquire increasing levels of CD138 and decreasing levels of CD45R/B220 to finally arrive at ASC with B220^neg^ phenotype, which are either CD138^int^ or CD138^high^ [32]–[33]. CD43 (leukosialin or sialophorin) is a cell surface sialoglycoprotein implicated in cell adhesion and proliferation whose tightly regulated expression in B lymphocytes is likely important for their normal development and/or function [34].

**Figure 6:**
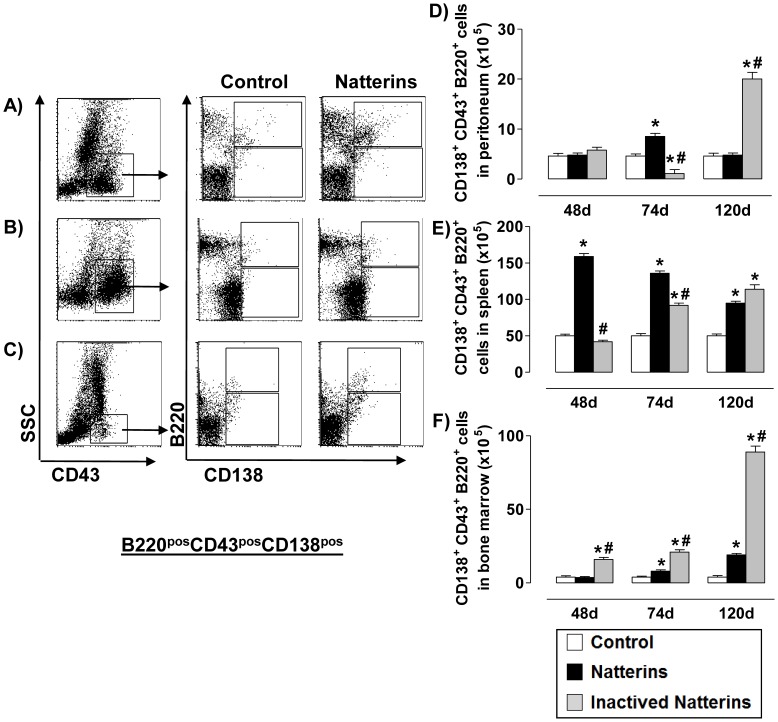
The early maintenance of ASC B220^pos^ and the exit from the peritoneal cavity and bone marrow are dependent on the proteolytic activity of Natterins. Total ASC B220^pos^ (B220^pos^CD138^pos^ from CD43-positive gated cells, upper box) numbers in peritoneum (***A***, ***D***), spleen (***B***, ***E***) and BM (***C***, ***F***) was determined from total mononuclear cells by multiparametric flow cytometer using Rat IgG2ak PerCP-Cy5-anti-mouse CD45R/B220, Rat IgG2ak FITC-anti-mouse CD43, and Rat IgG2ak PE-anti-mouse CD138. *p<0.05 compared to control-mice, and # *p*<0.05 compared to active Natterins.

**Figure 7:**
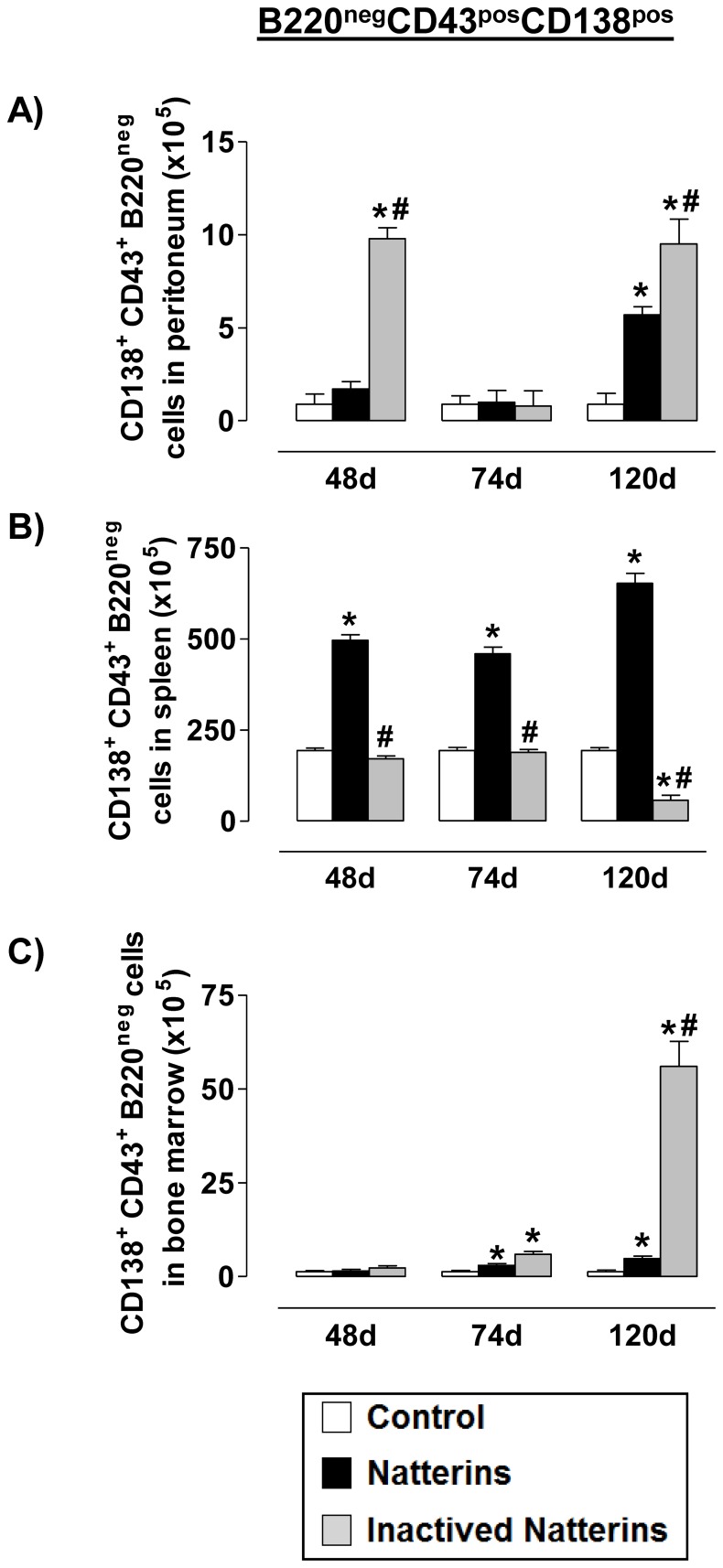
The proteolytic activity of Natterins induces the maintenance of ASC B220^neg^ in the spleen. Total ASC B220^neg^ (B220^neg^CD138^pos^ from CD43-positive gated cells, lower box) numbers in peritoneum (***A***, ***D***), spleen (***B***, ***E***) and BM (***C***, ***F***) was determined from total mononuclear cells by multiparametric flow cytometer using Rat IgG2ak PerCP-Cy5-anti-mouse CD45R/B220, Rat IgG2ak FITC-anti-mouse CD43, and Rat IgG2ak PE-anti-mouse CD138. *p<0.05 compared to control-mice, and # *p*<0.05 compared to active Natterins.

First, analyzing the levels of CD43 in ASC of all compartments we observed that the expression of this molecule is higher in cells from the spleen (Figures **6** ***B*** and **7** ***B***) compared with peritoneal cavity and BM. The high expression of CD43 molecule on splenic ASC may indicate a greater need of ASC to adhere to endothelial cells of the spleen, the ideal place to encounter antigens coming from the circulation and to maintain contact with the mediators required for ASC survival.

In the Figure **6**, we examined the population of ASC B220^pos^ (CD43^pos^CD138^pos^ with positive expression of B220) in cell suspension from animals immunized with active or inactivated Natterins. Our results show that the proteolytic activity of Natterins induced ASC B220^pos^ at 74 d in the peritoneal cavity (Fig. **6** ***A and 6D***), an elevated number in spleen until 120 d (Fig. **6** ***B and 6E***), and an expansion in the BM after 74 d (Fig. **6** ***C and 6F***). In addition, spleens were the chronic reservoirs of this cellular subpopulation in animals immunized with the active Natterins. The inactivation of proteases abolished the ability of Natterins to induce the influx to the peritoneal cavity and the expansion in the spleen at 74 d and, but in contrast inactivated Natterins mobilized elevated number of these cells at 120 d to the inflamed peritoneal cavity and to BM. These results indicate that the proteolytic activity of Natterins is related with the exit of the ASC B220^pos^ cells from the peritoneal cavity and from the BM.

In the Figure **7**, we examined the population of ASC (CD43^pos^CD138^pos^) with B220^neg^ phenotype in animals immunized with active or inactivated Natterins. We observed that spleen represent a favorable microenvironment for the prolonged maintenance of ASC with B220^neg^ phenotype in mice immunized with active Natterins. Peritoneal cavity and BM also contained at 120 d some ASC B220^neg^ compared with control-group of mice. The inactivation of protease activity abolished the ability of Natterins to sustain these cells in the spleen, and also retained these cells in peritoneal inflamed cavity and in BM. These results indicate that the protease activity of Natterins has an important role in driving the maintenance of ASC B220^neg^ in the spleen from the peritoneal cavity and from the BM.

Measurement of class switching is best done by staining for surface expression of the various IgH isotypes on activated B cells. For this purpose, we used saponin/trypsin treatment to effectively remove both membrane-bound and cytophilic IgE from activated B cell surfaces according to Wesemann et al. [22] and we investigated the percentage of intracitoplasmatic IgG- or IgE-positive ASC of both subtypes in all 3 compartments of Natterins-immunized mice at day 28, and our results demonstrated that: active Natterins induce IgG producing-ASC of both phenotype: B220^pos^ mainly in the spleen and B220^neg^ mainly in peritoneum and spleen. The inactivation of Natterins causes a decrease in the percentage of IgG producing-ASC B220^neg^, but sustains low percentage of B220^pos^ phenotype in all compartments, confirming that the stimulation with inactivated Natterins sustains the development of IgG producing-ASC B220^pos^. Also the active Natterins induce the IgE producing-ASC of both types: B220^pos^ in spleen and mainly B220^neg^ in all compartments. The inactivation of Natterins abolishes the percentage of IgE producing-ASC, demonstrating that B220^neg^ are responsible for the production of IgE (Fig. S**1**).

## Discussion

A number of environmental allergens from diverse sources have proteinase activity, which has been suggested to skew the immune response toward the Th2 phenotype. Long-lived antibody-secreting cells (ASC) are the source of IgE and recent data [1]–[3] suggest a role of IgE-producing splenic memory B cell and ASC in the development and maintenance of allergies. Although much is known about the cellular and molecular mechanism involved in the protease action that determine Th2 polarized responses [14], their direct or indirect actions in the regulation of the induction and survival of Ab-producing memory B cells or ASC in differential compartments remain largely unknown.

**Figure 8:**
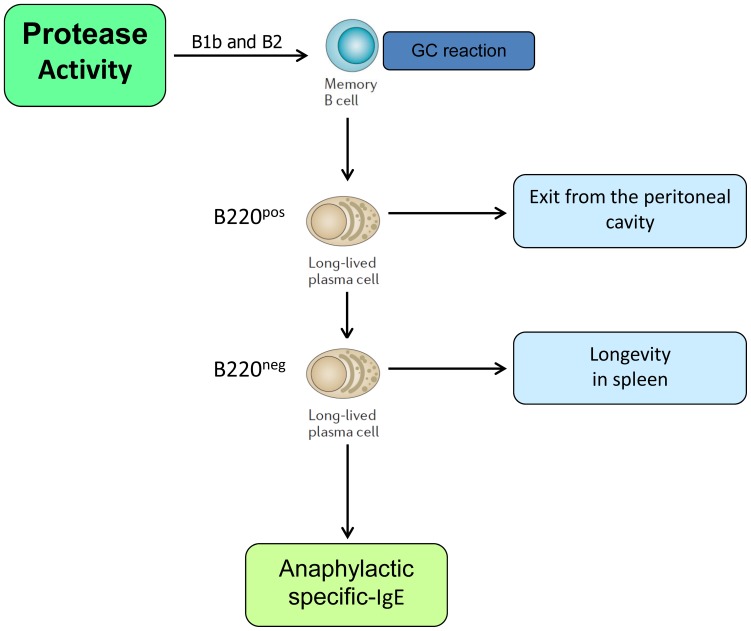
Possible scenario for the effect of Natterins on differentiation and survival of ASC in spleen. The development of long-term immunity to Natterins could be characterized by persistent anaphylactic-Abs derived from continuous differentiation in germinal center of Bmem from innate-like B1b and B2 cells and derived from linear differentiation of ASC B220^neg^ from B220^pos^ and Bmem.

In addition to the remaining challenge of determining in molecular detail how allergenic proteinases trigger allergic responses, in this study, we provide a new evidence that the Th2 polarized humoral response induced by proteases need a key component–the participation of long-lasting Bmem and ASC. Collectively, we showed that: (1) in the early phase of response against Natterins, the proteolytic activity positively controls the formation of B1b cells in spleen and BM, of Bmem in all compartments, of ASC B220^pos^ and ASC B220^neg^ in the spleen, and the production of Th2 Abs as anaphylactic IgG1 and IgE. Also in the early phase, the proteolytic activity of Natterins negatively controls the formation of B1a in the peritoneal cavity, of B2 in all compartments, of ASC B220^pos^ in BM and ASC B220^neg^ in peritoneal cavity; (2) in the chronic phase of memory response to Natterins, the proteolytic activity controls the exit of B2 and Bmem from the peritoneal cavity, and the exit of ASC B220^pos^ and ASC B220^neg^ from both niche peritoneal cavity and BM, inducing the maintenance of B1a and B2 in spleen and BM, and B1b, Bmem and ASC B220^neg^ in the splenic niche. Finally, high-affinity specific IgG1 and IgE Abs response dependent on the protease activity of Natterins is produced by memory B cells and ASC generated in the GC after innate-like B1b or B2 activation, but not by peripheral innate-like B cells that have not entered into GC (Fig. **8**).

The ability of proteases to induce potent Th2 humoral immune responses has long been known. Several cysteine and serine protease allergens have been cloned from house dust mites, including Der p 1, Der p 3, Der p 6, and Der p 9. A significant body of evidence suggests that these allergens mimic helper Th2 cell adjuvants. It is a consensus that proteases are allergenic not due to their structural features or class identity, but rather due to their proteinase activity. The pro-inflammatory effects of proteases have been attributed to their ability to augment mucosal permeability, to interact with protease activated receptors (PAR), to degrade tight junction proteins, to induce cytokine secretion and to cleave regulatory molecules present on various cells [35]–[37]. Furthermore, removal of proteases from either *A. fumigatus* [13], American cockroach Per a 10 antigen [38], Epi p1 antigen from the fungus *Epicoccum purpurascens* [36] or Cur 11 antigen from the mold *Curvularia Iunata* [39] decreases their ability to promote IgE Abs secretion, airway inflammation and airway hyperresponsiveness in mouse models.

Our data more importantly demonstrate the possibility that Th2 commitment, IgE switching and ASC with B220^neg^ phenotype arising from allergen exposure are ultimately driven by a protease-based mechanism, and we extend these findings including a new class of protease derived from venomous fish with kininogenase activity. Both Natterins (active and inactivated) induce the differentiation of the 2 subtypes of ASC: B220^pos^ and B220^neg^, however only the Natterins dependent on protease activity promote the production of specific-IgE and sustain the longevity of ASC B220^neg^ in the spleen (Fig. S**1** and Fig. **7** and **8**). Together, our data indicate a positive correlation between anaphylactic Abs production and terminally-differentiated end stage cells (ASC B220^neg^), extending the idea that beyond the maturity of B cell as an intrinsic B cell property that affects CSR choice of IgE versus IgG1, the splenic microenvironment activated by proteases also collaborates to class switching by the preservation a rich milieu for longevity of terminally differentiated cells.

B1 cells emerge early in development, are abundant in the peritoneal and pleural cavities, and are defined by their surface marker expression pattern as B220^low^IgM^high^IgD^pos^CD23^neg^. B2 cells are defined phenotypically as B220^high^CD23^high^. B1 cells are long-lived, self-renewing cells that produce poly-reactive IgM, referred as natural Abs, and do not undergo extensive somatic hypermutation. Both B1 cells and marginal zone B2 cells seem to be innate-like B cell populations that produce rapid but low-affinity IgM Abs to protect the host from pathogens entering through mucosal surfaces and the blood. Several reports confirm that Bmem and ASC can be generated during the first immune response from innate-like B cells as B1 and conventional B (B2).

The normal levels of B1a cells similar to control mice into peritoneal cavity observed during 48 to 120 d after immunization with active Natterins does not rule out that these cells have proliferated before, at early stage (28–48 d) of the chronic response as venom proteins did (19); and have migrated out of the peritoneum with subsequent differentiation into modest affinity-IgM-secreting ASC in lymphoid organs (e.g., the spleen) [6]. B1a cells quickly up-regulate the levels of Blimp-1 [40] and the process of differentiation is conducted outside of peritoneal cavity [41].

Activation-induced redistribution of body cavity B1 cells seems to be a common response in various disease models, it is probable that multiple innate immune signals can induce changes in B1 cell mobility, possibly by regulating their expression of integrins and chemokine receptors, although this remains to be fully explored. The participation of TLR and MyD88 signals in the traffic of B1 cells in mice immunized with Natterins is under investigation in our laboratory.

Moreover, it was hypothesized that B1 cell activation (which share similarities with CD5 human B cell) might also play a role during allergic immune responses, since these cells constitutively express the alpha chain of the IL-5 receptor - IL-5Rα/CD125 [42]–[44]. However, Kerzel and colleagues [45] described in a large sample of IgE transcripts from children with allergic asthma evidence of enhanced Ag selection, follows the traditional B2 adaptive B cell pathway. Our data agree with this human finding when demonstrated that the proteolytic active Natterins induce in mice specific-IgE production and a chronic splenic response of innate-like conventional B cells (B2) as well as B1 (B1a and B1b), but in contrast inactivated Natterins promoted the expansion only of B2 cells and IgG2a specific-Abs production. Our study identified that unlike B2 cells, the emergence of CD5+ B1 cells (B1a phenotype) seemed to be important for the control of the magnitude of Th2/IgE polarization.

Evidence supporting a B cell negative regulatory function has accumulated over the past 30 years. Dysregulated type 2 immunity in allergic asthma is characterized by an expansion of CD4^+^ Th2 cells and cytokines (IL-4, IL-5, IL-13), elevated total and allergen-specific IgE, eosinophilia, and airway inflammation and a counterbalance emergence of B1a cells with regulatory capacity [46]. Regulatory B cells are characterized by production of the negative regulatory cytokines, IL-10 and TGF-β. IL-10-producing regulatory B cells are of the CD19+CD5+IgM^high^IgD^low^CD1d^high^ type. However, the role of each of these innate-like B subsets remains elusive.

After T cell–dependent B cell activation, two sorts of plasma cells are produced. The first to produce Abs are short-lived memory B cells, which proliferate and differentiate in the lymph node and spleen; and the second are BM resident ASC which are generated mainly in the GC of peripheral lymphoid organs, as they show evidence of affinity maturation and selection [47]. ASC are terminally differentiated cells that no longer express surface-bound immunoglobulin but continuously secrete Abs without requiring further antigenic stimulation. The cellular differentiation of Bmem to ASC has not been completely elucidated, but a hierarchical model of differentiation has been proposed: activated B cells progressively acquire increasing levels of CD138 and decreasing levels of B220 to finally arrive at ASC with B220^neg^ phenotype, which are either CD138^int^ or CD138^high^ [33].

Our data here have shown that the proteolytic activity of Natterins drive the exit of Bmem and ASC B220^pos^ from the peritoneal cavity and from the BM, favoring the resting of ASC B220^neg^ in spleen. These findings confirm the existence in our model of a linear process of differentiation with terminal differentiation of ASC with B220^neg^ phenotype in splenic niche come from ASC with intermediate phenotype (B220^pos^) of inflamed peritoneal cavity and BM.

Furthermore, while the retention of Bmem in the inflamed tissue is negatively regulated by the proteolytic activity of Natterins, the maintenance in the BM in early stages, and mainly in the spleen for long-time, is totally dependent on this function. Finally, active Natterins preferentially control the prolonged survival of ASC with B220^neg^ phenotype in spleen. We proposed that Natterins rather than play a role augmenting memory B cell and ASC survival preventing apoptosis, they could direct act on B cell follicles of GC, up regulating homing receptors in resident mesenchymal stromal cells that influence migration and emigration or recycling of intermediate or high-affinity ASC. Future studies will address these possibilities.

Collectively, these data strongly support that proteolytic activity of Natterins is required for the final differentiation of Bmem into ASC with B220^pos^ phenotype and finally to B220^neg^ phenotype in GC where MMP-9 is also induced (data not shown), and more, drives the transit of ASC B220^neg^ from the BM and inflamed peritoneal cavity to the spleen. Thus, the immune system retains high-affinity memory responses while actively recruiting new memory B cells, thereby continuously diversifying the Abs response yet ensuring that high-affinity Abs are produced upon each Ag exposure.

Although active Natterins preferentially control the prolonged survival of ASC B220^neg^ and Bmem in spleen, the inactivated proteases in contrast promote the conservation of Bmem in peritoneal cavity and prioritize the differentiation of intermediate antibody-secreting cells in all compartments (ASC with B220^pos^ phenotype). Moreover, the absence of proteolytic activity due to inhibition by metal-chelating agents (EDTA and o-PHE) or by high temperature does not modify the capacity of Natterins to interact with APC for the induction of protective immunity. Natterins without proteolytic activity are also sufficient to induce a long-lasting immune response with high titers of IgG2a neutralizing Abs. Moreover, our study also confirms the role of inactivated Natterins as potent adjuvant.

Adjuvants are key components of vaccines and are selected on the basis of their effect on acute effector functions, such as induction of Th1, Th2, or Th17 T cell responses or their ability to promote certain Ig classes. However, long-term qualitative aspects of adjuvants–that is, their capacity to promote ASC or Bmem development have been insufficiently recognized [48]. The ability of inactivated Natterins to promote the preferentially localization of Bmem and of ASC with B220^pos^ phenotype in inflamed peritoneal cavity could be recognized as an adjuvant function for vaccines improvement, once this plasma cell subset accounts for the rapidity of a secondary response generating terminally-differentiated end stage cells (ASC B220^neg^). Our study contribute to the better understanding of the mechanisms imprinting tissue-specific migration onto Bmem as well as ASC with B220^pos^ phenotype and potential intersection with the complex mechanisms involved in the differentiation of ASC B220^neg^. The presence of survival niches in inflamed tissues and activated secondary lymphoid tissues is an efficient way of providing maximal amounts of specific Abs at the site of inflammation.

In conclusion, our data allowed us the further clarification of the polarized Th2 humoral memory response induced by proteases derived from venomous fish with kininogenase activity, and allowed the understanding of complex organization of memory B cell compartment, especially of the long-lasting subtype (ASC). We showed that the proteolytic activity of Natterins is sufficient for the generation of signals that contributed to the formation of a survival niche in the spleen, essential for longevity of innate-like B cells and long-lived antibody-secreting cells of B220^neg^ subtype.

## Supporting Information

Figure S1 **CD138pos-ASC B220pos or B220neg were analyzed for IgG and IgE switching using fixation and permeabilization with trypsin before staining for IgE and IgG.** Cell samples were obtained from active or inactivated Natterins-immunized mice. Both groups of animals killed at day 28 and peritoneal cells were recovered by peritoneal flushing. BM cells were isolated from femur bones and after centrifugation the supernatant from both cell suspensions were discharged. Spleen were removed aseptically and single-cell suspensions were prepared for Facs analysis. Cell were pelleted after surface staining (anti–B220-PE-Cy5, anti-CD138 APC) and resuspended with 1 ml of 3% formaldehyde for 20 min at 37°C, followed by a wash with 2 ml of PBS and permeabilization with 90% cold methanol for 30 min on ice, washed twice with PBS. The cells were pelleted and resuspended in 1 ml of 0.5% saponin or 0.05% trypsin for 2 min in PBS at room temperature and incubated for 1 h with a cocktail of PE anti-IgG, and FITC anti-IgE antibodies (both from BioLegend). Cells were then washed and re-suspended for flow cytometry.
(TIFF)
